# Design and characterization of a synthetic minimal promoter for heterocyst-specific expression in filamentous cyanobacteria

**DOI:** 10.1371/journal.pone.0203898

**Published:** 2018-09-11

**Authors:** Adam Wegelius, Xin Li, Federico Turco, Karin Stensjö

**Affiliations:** Department of Chemistry– Ångström Laboratory, Uppsala University, Uppsala, Sweden; Centre National de la Recherche Scientifique, Aix-Marseille Université, FRANCE

## Abstract

Short and well defined promoters are essential for advancing cyanobacterial biotechnology. The heterocyst of *Nostoc* sp. is suggested as a microbial cell factory for oxygen sensitive catalysts, such as hydrogenases for hydrogen production, due to its microoxic environment. We identified and predicted promoter elements of possible significance through a consensus strategy using a pool of heterocyst-induced DIF^+^ promoters known from *Anabaena* sp. PCC 7120. To test if these conserved promoter elements were crucial for heterocyst-specific expression, promoter-*yfp* reporter constructs were designed. The characterization was accomplished by replacing, -35 and -10 regions and the upstream element, with well described elements from the *trc* promoter of *Escherichia coli*, which is also functional in *Nostoc* sp. From the *in vivo* spatial fluorescence of the different promoter-*yfp* reporters in *Nostoc punctiforme* ATCC 29133, we concluded that both the consensus -35 and extended -10 regions were important for heterocyst-specific expression. Further that the promoter strength could be improved by the addition of an upstream element. We designed a short synthetic promoter of 48 nucleotides, P_synDIF_, including a consensus DIF1 sequence, a 17 base pair stretch of random nucleotides and an extended consensus -10 region, and thus generated the shortest promoter for heterocyst-specific expression to date.

## Introduction

Cyanobacteria are promising as platforms for biological production of fuels and other chemicals. Their fast growing photosynthetic nature makes them ideal from a sustainable production perspective. Within the vast and diverse cyanobacterial phylum, heterocyst forming cyanobacteria is a group of fascinating multicellular photosynthetic organisms that are able to differentiate a subset of their cells into specialized compartments dedicated to fixation of atmospheric nitrogen, called heterocysts. The interior of the heterocyst is kept micro-aerobic to let the oxygen sensitive nitrogenase, the enzyme-complex responsible for the nitrogen fixation, operate. The development of the nitrogen fixing cells is a complex process where environmental stimuli and a large number of signaling substances interact and give raise to the characteristic, semi-regular pattern of heterocysts along the filament. The process starts when no source of combined nitrogen is available and is coordinated by the global nitrogen transcription regulator NtcA [1]. Another key factor in the differentiation process is the regulator HetR, which is activated by NtcA. HetR regulates a large number of genes involved in the differentiation process and is known to be a major regulator in heterocyst development [2–4]. The differentiation of a vegetative cell into a heterocyst results in drastic metabolic changes as well as noticeable morphological alterations, such as the development of a thick cell envelope outside the outer membrane [1,5–8].

Heterocysts are interesting for production of biofuels and chemicals, as the unique cellular environment should be well suited for heterologous expression of oxygen sensitive enzymes, like hydrogenases [9–11]. To spare the cell from unnecessary metabolic burden caused by expression of proteins within a non-suitable environment, it is crucial to have tools to express a given protein solely in heterocysts. Up to this point, all heterocyst-specific metabolic engineering approaches have been utilizing native and often very long and poorly characterized promoter sequences found upstream of heterocyst expressed genes. Examples of this are the heterocyst-inhibiting signaling peptide (PatS)-promoter [12], the *alr3808*-promoter [13,14], the *hepA*-promoter [15] from *Anabaena* (*Nostoc)* sp. PCC 7120, and truncated versions of the uptake hydrogenase promoter from *Nostoc punctiforme* ATCC 29133 (*N*. *punctiforme*) [16].

The usage of native, often several hundred nucleotides long, promoter sequences in biotechnological applications is not optimal. Such native sequences are often heavily regulated and part of the complex and multi-layered internal metabolic regulatory system, which can give rise to unpredictable behaviors and unforeseen effects in the expression system. Naturally, long pieces of DNA are also less convenient when the expression constructs are being assembled, especially for large and more complicated constructs.

The shortest promoter known to render heterocyst-specific expression of heterologous genes is the 70 nucleotides long native *nsiR1*-promoter (*P*_*nsiR1*_) from *Anabaena* sp. PCC 7120 [14]. This promoter controls the transcription of the *nsiR1* (nitrogen stress inducible RNA1), a sRNA induced early in heterocyst development [17]. *P*_*nsiR1*_ is well studied and has been used in a promoter-fluorescence reporter system in *Anabaena* sp. PCC 7120 for detection of proheterocysts before any morphological change specific to heterocyst are visible with microscopy [18]. The *P*_*nsriR1*_, as well as *alr3808*-promoter, belongs to the to the DIF^+^ class promoters [14]. This is a family of promoters associated with heterocyst-specific expression and contains the DIF1-motif [19] (consensus sequence 5’-TCCGGA-3’) located at the -35 position relative the transcription start site (TSS).

The native *P*_*nsiR1*_ is shorter than many other promoters used for heterologous expression in cyanobacteria, but still not as well defined as promoter sequences commonly used in synthetic biology approaches in more traditional industrial organisms like *Escherichia coli* [20] and yeast [21]. To improve and expand the metabolic engineering toolbox, and to address the lack of minimal synthetic promoters for heterocystous cyanobacteria, we have in this work created the shortest heterocyst-specific promoter to date. The promoter was constructed according to synthetic biology principles using consensus architecture design [22]. This is a well-defined, minimal promoter inspired by natural DIF^+^ class promoters. The synthetic nature of this promoter ensures that is can be used without risk of unknown native regulation and due to its short length, it is efficient to use in cloning processes and construct assembly.

## Results and discussion

### Design of a compact synthetic DIF^+^ promoter

**Fig 1:**
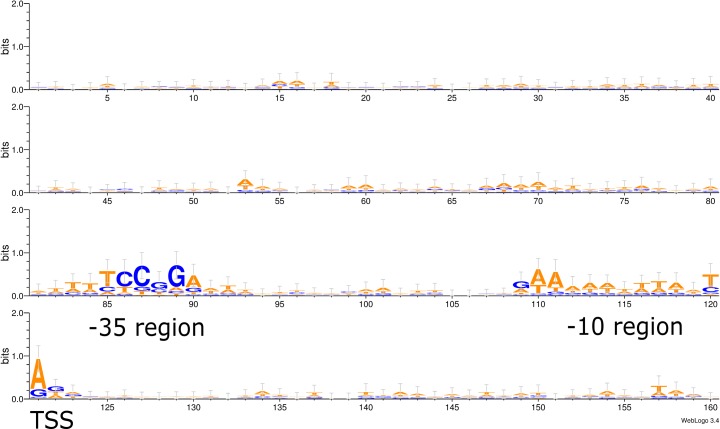
Consensus sequences of -35 and -10 elements of 58 DIF^+^ promoters. Alignment of 58 DIF^+^ promoter sequences, from 40nt downstream to 120nt upstream of TSS, identified by Mitschke *et al* [14]. The result is presented by the weighted sequence logo (WebLogo 3.0) [23] and conserved regions at -35 and -10, as well as the transcription start site (TSS) are indicated.

**Fig 2:**
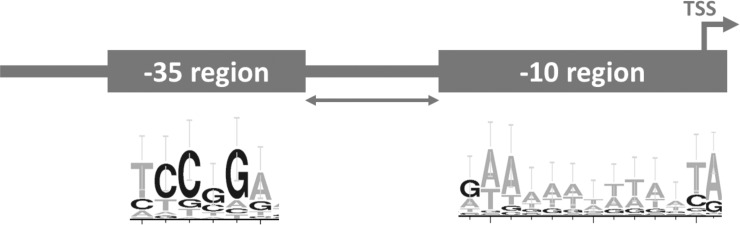
Schematic representation of the promoter elements of the minimal synthetic promoter, P_synDIF_. Transcription start site (TSS), extended -10 region, spacer region (arrow) and -35 region are indicated. Consensus sequences from the alignment of 58 DIF^+^ promoters used in the design of the minimal promoter are depicted as WebLogos [23] below the promoter.

We aligned 58 DIF^+^ class promoter regions from *Anabaena* sp. PCC 7120 [14] and graphically represented it as a WebLogo [23] (Fig 1). Apart from the already reported DIF1 motif at position -35 (relative TSS) [19], we found a highly conserved AT-rich sequence pattern around the -10 region and TSS. This conserved sequence differs from the classical, SigA-associated -10 region (5’-TATAAT-3’). The alignment revealed no conserved patterns upstream -35 or between the -35 and -10 regions. We propose that the conserved heterocyst enhanced expression pattern from the DIF^+^ class promoters should be an effect of conserved regions in the promoter sequences. The lack of conserved sequence patterns upstream the DIF-motif suggests that a minimal promoter sequence could contain all genetic information needed for heterocyst-specific expression. Based on our observations, we designed a synthetic, 48 nucleotides long, ultra-compact promoter, P_synDIF_, by combining the DIF-motif at position -35 with the further downstream consensus sequence from the alignment, making up an extended -10 region, spanning from -13 to TSS (Fig 2). To minimize the risk of including unknown regulation, the bases in the spacer region between the -35 and -10 regions were chosen randomly, but the GC-content and number of nucleotides were kept identical to the corresponding region in the heterocyst-specific *NsiR1*-core promoter from *Anabaena* sp. PCC 7120 [14,17,18]. Upstream the DIF1 motif, 12 nucleotides were put as spacer between promoter and the plasmid backbone.

### Expression pattern of short synthetic DIF^+^ promoters in *N*. *punctiforme*

To elucidate if our minimal synthetic DIF^+^ promoter had retained the heterocyst-specific expression pattern typical for the native DIF^+^ promoters, we designed a promoter fluorescence reporter construct for *in vivo* detection. The P_synDIF_ was put upstream of the gene *eyfp*, which encodes a yellow fluorescent protein (YFP), together with the cyanobacterial synthetic ribosomal binding site RBS* (“RBSstar”) [24] in the self-replicating shuttle vector pSAW_yfp_ (created in this work, see Materials and methods and S1 Fig), resulting in the SynDIF-pSAW plasmid. To be able to resolve the importance of the consensus regions in the synthetic promoter, we developed two similar constructs where the -35 and -10 regions of the P_synDIF_ were respectively exchanged to the corresponding regions from the P_*trc1O*_ promoter, with a SigA-typical -10 region, known to be constitutively expressed in heterocysts and vegetative cells of *N*. *punctiforme* [25]. The two promoters were included in fluorescence reporter plasmids similar to SynDIF-pSAW, resulting in plasmids -10P_*trc*_-SynDIF-pSAW and -35P_*trc*_-SynDIF-pSAW. The three plasmids, as well as a promoterless control plasmid, were electroporated into *N*. *punctiforme*.

**Fig 3:**
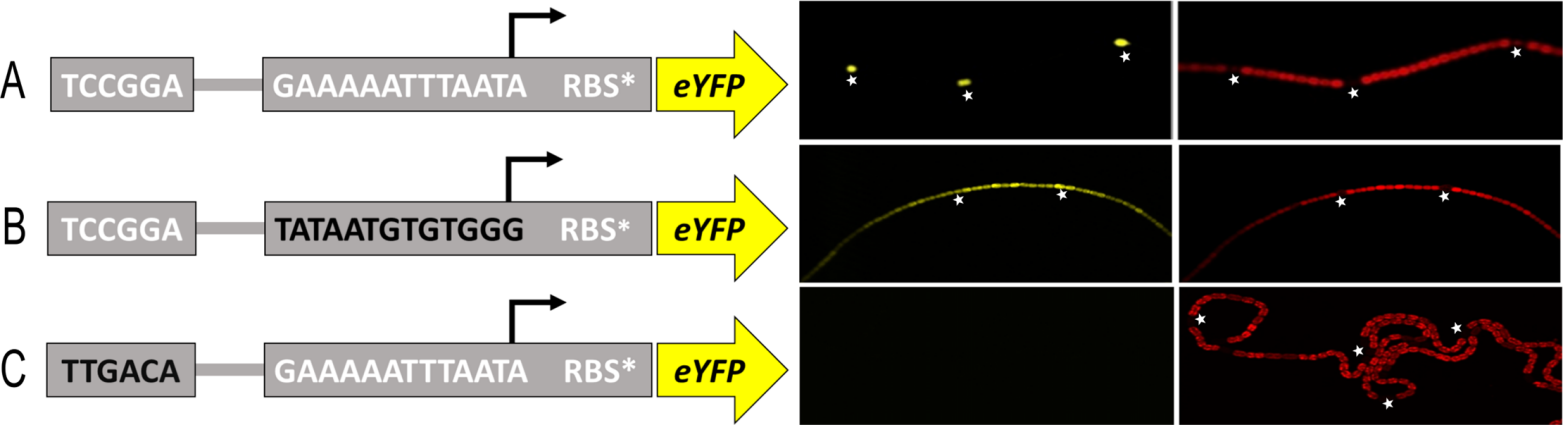
Design of SynDIF reporter constructs and YFP expression along filaments of *Nostoc punctiforme* 12 hours after removal of combined nitrogen. Schematic representations of the synthetic promoter-reporter constructs, (A) SynDIF, (B) -10P_trc_-SynDIF and (C) -35P_trc_-SynDIF are shown to the left. Black colored letters indicates bases changed from the SynDIF. Representative confocal fluorescence images of *N*. *punctiforme* filaments are shown with fluorescence from the three promoter-YFP constructs in yellow (530-540nm), and autofluorescence in red (600-700nm). Heterocysts, identified by the reduced autofluorescence, are indicated by stars.

After 12 h of combined nitrogen starvation, the expression patterns from the three promoter variants were investigated by fluorescence confocal microscopy. Representative filaments from each strain were imaged and can be seen in Fig 3. The strain bearing the SynDIF-pSAW plasmid (SynDIF strain) showed clear YFP fluorescence restricted to heterocysts, establishing our minimal synthetic promoter as heterocyst-specific in *N*. *punctiforme*. After 24 h of nitrogen starvation, twenty heterocysts from three individual cultivations of the SynDIF strain were identified by morphology and reduced autofluorescence. In a second step, these heterocysts were investigated for YFP fluorescence. All of the investigated heterocysts, in total 60, were confirmed to have drastically higher fluorescence then the 5 closest cells on each side (S1 File), similar to the representative picture in Fig 3. An overview image of multiple filaments can be found in supporting information, S2 Fig. The P_synDIF_ is, to the best of our knowledge, the shortest promoter ever described to provide heterocyst-specific expression of a reporter gene and its fully synthetic nature makes it truly unique. In the strain bearing the -10P_*trc*_-SynDIF-pSAW plasmid (SynDIF-10P_*trc*_ strain), distinct YFP fluorescence could be seen in both heterocysts and vegetative cells with no distinguishable difference in fluorescence level between the two cell types. It is thus clear that a DIF1-motif in the -35 position is not by itself enough to render heterocyst-specificity to a promoter sequence. Our result highlights the importance of the -10 region for the heterocyst-specific behavior of DIF^+^ promoters. The strain harboring the -35P_*trc*_-SynDIF-pSAW plasmid (SynDIF-35P_trc_ strain) did not display any detectable YFP fluorescence in any cells, indicating that the combination of the -35 region from P_*trc*_ and our consensus extended -10 region does not serve as a functional promoter in *N*. *punctiforme*. It is apparent that both the DIF1 palindrome and an appropriate -10 region are crucial for the heterocyst-specific expression pattern from our synthetic DIF^+^ promoter. Filaments bearing the promoterless control construct did not exhibit any detectable fluorescence. When *N*. *punctiforme* is grown in ammonium supplemented media, the formation of heterocysts is prevented, and under this condition the SynDIF strain showed only very low, irregular fluorescence from YFP. This is in agreement with what has been reported earlier for native DIF^+^ promoter-reporter constructs [18].

It has been shown previously, using native P_*nsiR1*_, that if the DIF1 palindrome of a DIF^+^ promoter is exchanged with a restriction site, the heterocyst-specificity is lost [14]. It is not unlikely that exchanging the -35 region of a promoter for a restriction site would severely affect the binding properties of the RNA-polymerase and therefore repress the activity of the promoter. This was the case when we changed the DIF palindrome into a sequence known as a functional -35 region in P_*trc*_. No detectable increase in fluorescence compared to the promotorless control was detected in the SynDIF-35P_trc_ strain upon nitrogen depletion (Fig 3). For us, this indicated a nonfunctional promoter and the conclusion that the DIF palindrome was solely the region needed for the heterocyst-specific expression pattern could not be drawn from this experiment. Indeed, our results show that also the -10 region plays a vital role for the expression pattern of DIF^+^ promoters.

The recognition of specific promoters and initiation of transcription are directed by sigma factors, which form complex with RNA polymerase [26]. Heterocyst development is controlled by a complex transcriptional regulon, in which at least three sigma factors, SigC, SigE and SigF have critical roles [27,28]. Due to the found importance of the conserved -35 and -10 regions of the SynDIF promoters for heterocyst specific expression, it is tempting to propose that one of these sigma factors might be the activator of DIF1 motif promoters. However, there are without doubt also possible candidates among the other ten sigma factors in *N*. *punctiforme* [29] and among the various other transcriptional regulators involved in heterocyst development and metabolism [30].

Based on our results we suggested that the heterocyst specific fluorescence of the SynDIF strain, detected at 12 hours after combined nitrogen depletion, was caused by an increase of *eyfp* transcription. However, the mechanism behind the increased level of fluorescence was not revealed by our experiment. Still there were questions if the intense fluorescence originated from a lasting enhancement of transcription from the P_synDIF,_ or from a transient activation of the promoter at an earlier stage of heterocyst differentiation, that could appear as a strong fluorescence signal at 12 hours after combined nitrogen depletion due to an accumulation of YFP.

**Fig 4:**
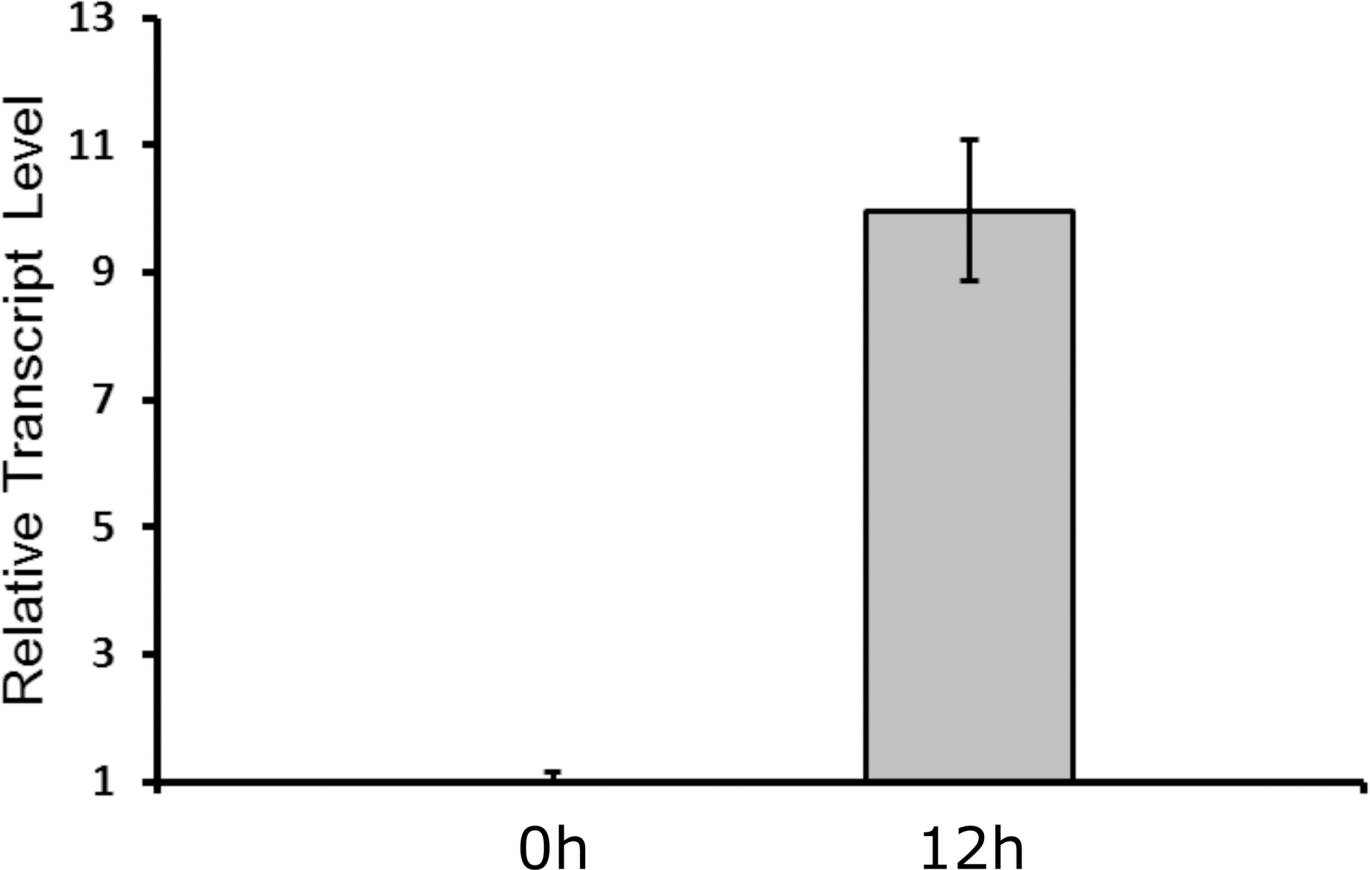
RT-qPCR analysis of the *eypf* transcript levels in *Nostoc punctiforme* engineered strain SynDIF at 0 and 12 h after nitrogen step down. Fold change in transcripts levels are shown as grey bars. Values are normalized to the 0h *eyfp* transcript level and *16S* ribosomal RNA was used as reference gene. Fold change in transcript level was calculated by using CFX manager® (Bio-Rad) software, which is based on the 2^-ΔΔCt^ method [32]. Three biological and three technical replicates were used for each sample and error bars indicate standard error of mean from biological replicates.

To closer investigate the expression from P_synDIF_ upon nitrogen step-down, the transcript level of *eyfp* was investigated by RT-qPCR. Twelve hours after nitrogen depletion of the SynDIF, a 10 fold increase in *eyfp* mRNA levels compared to the levels at 0 h was observed (Fig 4). This result suggest that the observed increase of YFP in heterocyst is not a result of an early transient expression of mRNA resulting in high levels of the YFP protein 12 h after nitrogen depletion, but indeed of a lasting increase in mRNA level. Heterocyst-specific fluorescence from a P_DIF-native_-GFP strain of *N*. *punctiforme* was previously reported as early as five hours after nitrogen step-down [13]. Also, the transcript of a gene regulated by the native *nsiR1*-promoter, was detected as early as three hours after nitrogen-depletion and the abundance level of this transcript was constant from three to 12 hours after nitrogen depletion [18]. To investigate the YFP expression from the P_synDIF_ throughout the development process, single filaments were monitored by confocal microscopy from the time of nitrogen deprivation and 48 h forward. Images of a representative filament at 6, 12, 24 and 48 h can be seen in supplementary information (S3 Fig). At 48 h, heterocysts were still showing clear YFP expression. The YFP variant used in this work (EYFP, see Materials and methods) has been established to have an *in vivo* half-life of 12.8 h in a cyanobacterial host [31]. The limited *in vivo* half-life, together with the unmistakable YFP fluorescence in mature heterocysts 48 h after nitrogen deprivation, is a clear indication that the P_synDIF_-activity is maintained also in later stages of heterocyst differentiation, confirming its usefulness for biotechnical applications.

### Altering expression level by addition of native upstream region

**Fig 5:**
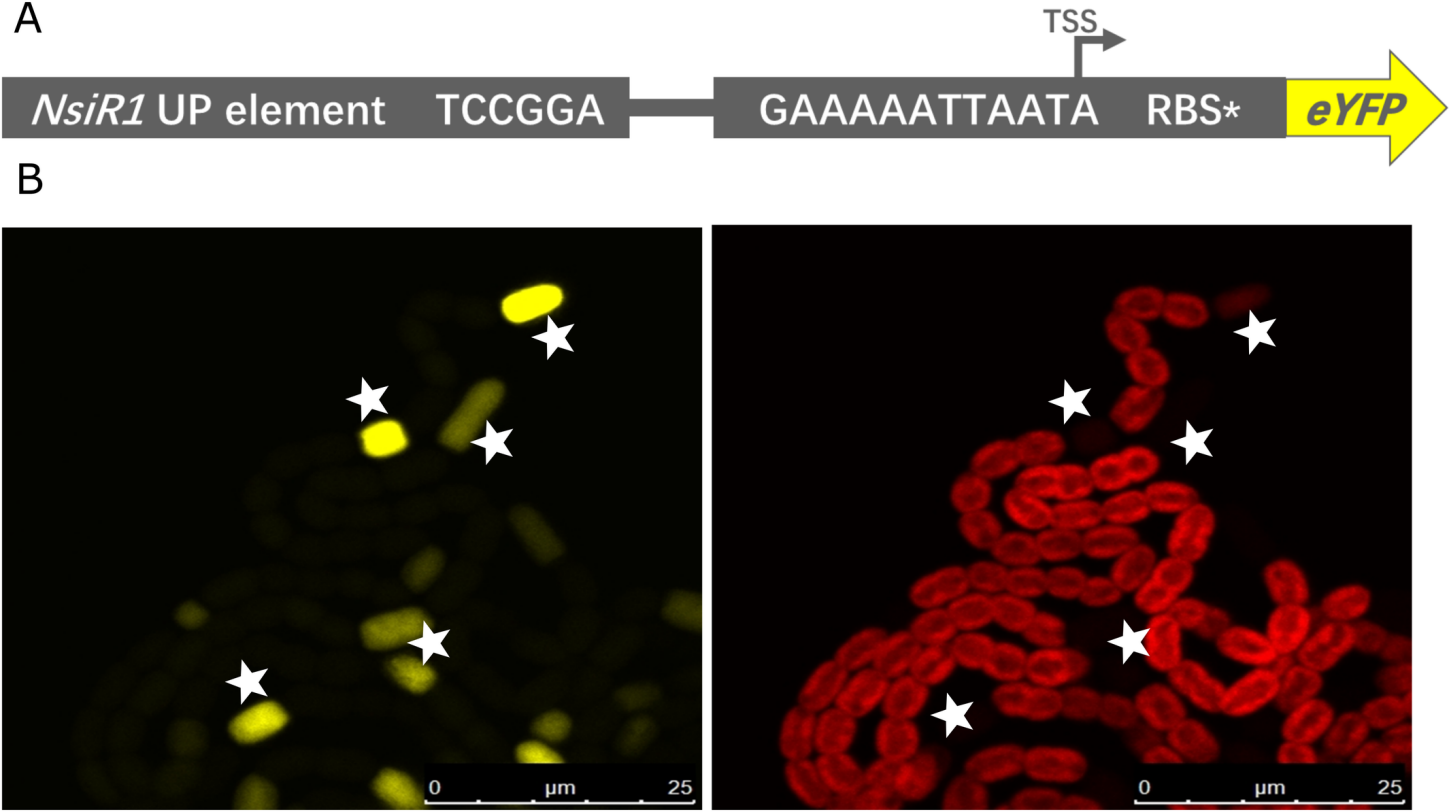
Design of the UESynDIF reporter construct and YFP expression along filaments of *Nostoc punctiforme* 12 hours after removal of combined nitrogen. (A) Schematic representation of the P_UEsynDIF_-EYFP construct. P_UEsynDIF_ is a version of the P_synDIF_ with an additional upstream element (UP element) from the native *Anabaena* sp. PCC 7120 *nsiR1*-promoter. (B) Representative confocal fluorescence images of a *N*. *punctiforme* filaments carrying the UESynDIF-pSAW plasmid. Fluorescence from YFP (530-540nm) is shown in yellow (left) and autofluorescence (600-700nm) is shown in red (right). Heterocysts with reduced autofluorescence are indicated by stars.

**Fig 6:**
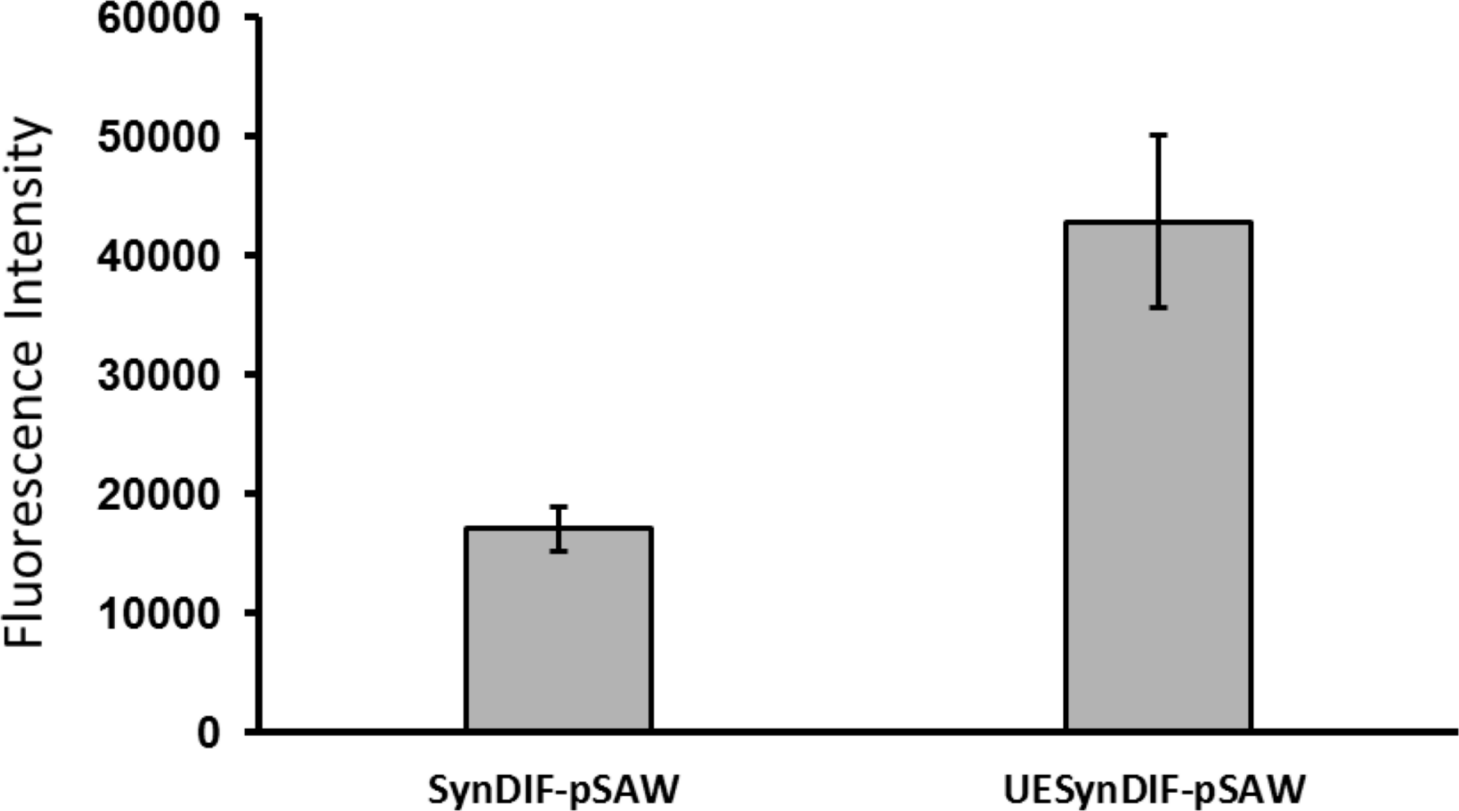
Quantitative analysis of heterocyst-specific promoter activities in *Nostoc punctiforme* cultures, 12 hours after removal of combined nitrogen. Mean value of YFP fluorescence intensity per heterocyst in strains carrying SynDIF-pSAW and UESynDIF-pSAW plasmids. YFP fluorescence intensity data acquired from Merck Amnis FlowSight was analyzed using IDEAS software. The data represent mean ± SD of triple measurements of three independent cultivations.

The sequence immediately upstream the -35 region is known to be important for transcription initiation and regulation. To study the effect of an upstream element (UE) on our synthetic minimal promoter, we chose a stretch of 35 base pairs upstream the DIF1 motif in the native *nsiR* promoter and employed it immediately upstream the DIF1 motif in P_synDIF_. The resulting native/synthetic-hybrid promoter, P_UEsynDIF_ (Fig 5A), was utilized in the same reporter construct as the minimal promoters, resulting in the UESynDIF-pSAW plasmid. After transformation into *N*. *punctiforme*, filaments were investigated for cell specific fluorescence. Also in this strain, YFP fluorescence was observed in heterocysts 12 h after combined nitrogen depletion (Fig 5B), confirming the function and cell specificity of the native/synthetic-hybrid promoter. Above 90% (91.7% average between three independent cultivations) of the heterocysts in this strain where confirmed to have drastically higher YFP fluorescence then the 5 closest cells on each side 24 h after nitrogen starvation (S1 File). The fluorescence level in fluorescing heterocysts from the hybrid promoter strain, as measured with quantitative flow cytometry, was significantly higher than the fluorescence from the SynDIF minimal synthetic promoter construct (Fig 6). This result, together with the confocal data, indicates that an upstream element can be utilized to alter the level of heterocyst-specific accumulation of YFP-protein from DIF^+^ promoter constructs. In the case of our minimal synthetic promoter, we show that the addition of an upstream element can enhance the expression level while the heterocyst-specificity is, to a high degree, preserved.

## Conclusions

In this work we use consensus sequences of core promoter elements to build a minimal synthetic promoter for heterocyst-specific expression. This promoter gives an approximate 10 time increase in expression 12 hours after the start of heterocyst differentiation. This well characterized promoter expands the potential for cyanobacterial biotechnology and is of special importance for expression of oxygen intolerant enzymes in a photosynthetic host organism. This work provides fundamental understanding of the promoter elements of a core promoter and adds to the knowledge concerning the importance of the cooperation of -35 and -10 regions, and the usefulness of upstream element to enhance the expression level. From recent work by Elhai and Khudyakovit [33], it can be concluded that the DIF1 motif is conserved in the upstream regions of genes involved in heterocyst differentiation in a large number of cyanobacteria. Although the cell specificity of DIF^+^ promoters have only been investigated in *Anabaena* sp. PCC 7120 and *Nostoc punctiforme* ATCC 29133, the SynDIF promoters are of potential interest for heterocyst-specific protein expression in cyanobacteria beyond these two model strains.

This work has expanded the available toolbox for heterocyst-based biotechnology. The developed promoters will be especially useful in bio-hydrogen production from heterocystous cyanobacteria, an area of importance in the search for sustainable energy-carriers for our society.

## Materials and methods

### Promoters and plasmids construction

All oligonucleotide primers used in this work are listed in Table 1. The plasmid pSAW was created in this work (S1 Fig). The plasmid fragment responsible for kanamycin resistance and self-replication in *Nostoc punctiforme* and *Escherichia coli* was amplified from pSCR119 [34] with 5’-phosphorylated primers SCR_F and SCR_R. Flanking BioBrick [35] terminators BBa_b0052 and BBa_b1007 was introduced as overhangs on the primers. The resulting fragment was blunt end ligated to a DNA fragment containing a ccdB-cassette [36] flanked by restriction sites (ScaI, XbaI and SalI upstream, XhoI, PstI and KpnI downstream) resulting in a self-replicating intermediate plasmid, pSAW_ccdb_ (S1 Fig). The ccdB fragment was obtained by PCR amplification with primers ccdb_F/R using 50 ng of pPMQAK1 [25] as template. An *eyfp* gene with terminator was amplified with primers Eyfp_F/R using P_trcO_1-eyfp-bbaB0015-pSB1AC3 plasmid [25] as template, and transferred to the intermediate plasmid by restriction digestion using PstI and XhoI, resulting in the pSAW_yfp_ vector (S1 Fig).

**Table 1:** Oligonucleotide primers used in this study.

Primer name	Primer sequence (5’-3’)	Plasmid/Experiment
SCR_F	cgcaaaaaaccccgcccctgacagggcggggttttttcgcgtgccagctg-cattaatgaatcggccaa	pSAW
SCR_R	agaaatcatccttagcgaaagctaaggattttttttatctgaattcttttgttatat-cggcggaaagctttgag	pSAW
ccdB_F	gagctctctagacccggggtcgacactggctgtgtataagggagcct	pSAW
ccdB_R	ggtacctgcagcccgggctcgagacgcgtggatccggcttactaaa	pSAW
Eyfp_F	atcgctcgagatggtgagcaagggcgagga	pSAW
Eyfp_R	cgatctgcagtataaacgcagaaaggcccacc	pSAW
DIF-10trc_F	gggtgtgtaatatatttgatagatggatagag	-10P_trc_-SynDIF-pSAW
DIF-10trc_R	cacacccatcctaggatcacctccaga	-10P_trc_-SynDIF-pSAW
DIF-35Ptrc_F	acagataggtagatagtttagaaa	-35P_trc_-SynDIF-pSAW
DIF-35Ptrc_R	caaaaaacaatgcgttctaga	-35P_trc_-SynDIF-pSAW
YFP_qPCR_F	gctaccccgaccacatgaag	qPCR experiment
YFP_qPCR_R	gatgcccttcagctcgatg	qPCR experiment
16S_qPCR_F	gaataagcatcggctaactccg	qPCR experiment
16S_qPCR_R	ctacaccaggaattccctctgc	qPCR experiment

The P_synDIF_-RBS* and P_UEsynDIF_-RBS*, sequences were synthetized by GeneScript (Hong Kong) and inserted in pSAW by restriction/ligation using XhoI and XbaI, creating the plasmids SynDIF-pSAW and UESynDIF-pSAW. For the full sequence of the synthesized sequences, see supporting information (S2 File). The plasmids -35_Ptrc_-SynDIF-pSAW and -10P_trc_-SynDIF- pSAW were generated by Site Directed Mutagenesis using PCR amplification with outward-facing, non-overlapping 5’-phosphorylated primers followed by blunt end ligation. The plasmid SynDIF-pSAW was used as template for the PCR reactions and the primers used for the respective plasmids are listed in Table 1. The promoterless YFP-plasmid used as control was created by restriction of pSAW_yfp_ with SalI and XhoI, cutting away the ccdB-gene while creating complementary overhangs, followed by ligation.

### Strains and growth conditions

The plasmids enlisted in the section “Promoters and plasmids construction” were transformed in *E*. *coli* DH5α or *E*. *coli* DB3.1 (when ccdB resistance was required) by standard procedure for conservation purposes and were transformed in *Nostoc punctiforme* ATCC 29133 by means of electroporation as described elsewhere [16], obtaining the strains SynDIF, UESynDIF, SynDIF-35P_trc_, SynDIF-10P_trc_ and promoterless-YFP. These, were grown in 100 mL Erlenmeyer flasks under 40 μmol photons s^-1^ m^-2^ of light at 25°C with gentle agitation. BG11 supplemented with 2.5 mM NH_4_, 5 mM HEPES and 25 μg/mL kanamycin was used for growth under normal conditions. For nitrogen step-down experiments, the cells were harvested by centrifugation at 3500 rpm and resuspended in BG11_0_ three times prior to a final resuspension in BG11_0_ supplemented with 25 μg/mL kanamycin.

### Confocal microscopy

The *N*. *punctiforme* cultures where imaged using a Leica DM600 CS microscope and a HPX PL Fluotar 40.0x0.75 dry objective. Samples were excited using a 488nm laser and emission was detected between 530nm to 540nm (EYFP channel) and between 600nm and 700nm (autofluorescence channel). Prior to imaging, the different strains of *N*. *punctiforme* were fixed on solid media by spreading 20 μL of cell suspension onto 50 mL petri dish filled with BG11_0_ 0.8% agarose media with kanamycin 25 μg/mL. Z-stacks and 3D-projections were acquired using Lecia Application Suite Advanced Fluorescence (LAS AF) software, according to manufacturer’s instructions using recommended settings.

### RT-qPCR

Triplicate cultures of the SynDIF strain and promotorless control strain were grown in 400 mL of BG11 medium, supplemented with 2.5 mM NH_4_, 5 mM HEPES and 25 μg/mL kanamycin, at a light intensity of 40 μmol photons s^-1^ m^-2^ at 25°C to an OD_750nm_ of 0.6. The cells were harvested at 0 and 12 hours and washed. RNA was isolated as previously described [37]. The RNA quality and concentration were analyzed with the Experion System (Bio-Rad, Hercules, CA, USA) according to the manufacturer’s instructions. Prior to RT reactions, RNA was treated with DNaseI (Thermo Fisher Scientific), and 1 μg of total RNA was converted to cDNA using qScript cDNA synthesis kit® (Quantabio) according to the manufacturer’s protocol. Melting curve, primer efficiency and expression test were performed by using CFX Connect^TM^ qPCR machine (Bio-Rad). YFP and 16S were used as target and reference genes respectively. Primer efficiency value was considered to be reliable for further expression experiments if ranging between 95% and 110% [38]. Fold change in expression level was calculated by using CFX manager® (Bio-Rad) software, which is based on the 2^-ΔΔCt^ method [32]. Three biological and three technical replicates were used for each sample. All primers used are listed in Table 1.

### WebLogo

Sequences for 58 DIF^+^ promotors with the DIF1 palindromic sequence 5’-TCCGGA-3’ (one mismatch allowed) located close to the −35 were obtained from [14]. Sequences, stretching from 40 nt downstream to 120 nt upstream of the transcription start site of respective DIF^+^ promoters, were aligned and the consensus sequences were generated and visualized by WebLogo 3.0 [23].

### Cell preparation for imaging flow cytometry, Flowsight ^®^

*N*. *punctiforme* strains SynDIF and UESynDIF were cultivated in BG11_0_ medium. 5 mL of culture (at an OD_750nm_ of approximately 1.0) was collected by centrifugation 12 hours after nitrogen step-down. Cells were resuspended in 2 mL of dH_2_O and sonicated at 40 W, for 1 min, and then cooled in ice-cold water. The samples were analyzed under microscope to ensure that the majority of filaments were ruptured into single cells. The samples were then centrifuged at 1,000 x g, for 3 min, at 4°C, to remove cell debris. Following centrifugation, cells were resuspended in 2 mL dH_2_O and incubated at RT.

### Quantitative study of heterocyst-specific YFP fluorescence

A minimum number of 10,000 cells were sampled on an image-based flow cytometer (Merck Amnis FlowSight) and analyzed using analysis software IDEAS. Single cells were identified based on the first gating with area scatter and aspect ratio scatter parameters. Thereafter, the cells were sub-gated with intensity scatter on YFP fluorescence channel, at 488nm channel (for YFP, FITC, AF488, and GFP) isolating all cells displaying fluorescence in the said channel. In order to isolate heterocysts, in case any other cells were displaying fluorescence in the YFP channel, a third step gating was performed. Based on the 488nm channel data, the cells were sub-gated with the intensity scatter on 745-800nm channel, indicating autofluorescence. The targeted single heterocyst cells were selected based on significantly decreased autofluorescence compared to vegetative cells.

## Supporting information

S1 Fig pSAW plasmid maps. Overview map of the plasmids pSAWccdb and pSAWyfp created in this work. Plasmid backbone contains kanamycin/neomycin resistance gene *npt* and origins of replication for *Nostoc punctiforme* (pDC1) and *Escherichia coli* (ColE1) from pSCR119 [34]. Toxin encoding *ccdB* gene for plasmid maintenance and selection in *Escherichia coli* [36] is expressed from the *ccdB*-operon. Terminators (brown) are named after their respective Parts Registry association number [35]. Recommended restriction sites for cloning are indicated.
(TIF)

S2 Fig Overview confocal fluorescence image of multiple filaments of *Nostoc punctiforme* SynDIF strain. 3D-projection of a Z-stack containing 91 slices of a 30μm deep sample volume. Fluorescence images from YFP channel (530-540nm) and autofluorescence channel (600-700nm) are overlaid.
(TIF)

S3 Fig Monitoring of single filament during heterocyst development process. Confocal fluorescence images of a representative filament of the SynDIF strain at 6, 12, 24 and 48 h after nitrogen deprivation immobilized on agar plate. Autofluorescence (600-700nm) is shown in red, YFP fluorescence (530-540nm) in yellow. Rightmost column shows overlaid images. In the autofluorescence images, the arrows indicate a developing heterocyst.
(TIF)

S1 File Investigation of YFP fluorescence in identified heterocysts of the SynDIF and UESynDIF *Nostoc punctiforme* strains. (DOCX)

S2 File Sequences of the promoter-RBS constructs. Full sequences of the promoter-RBS designs synthesized for this study.
(DOCX)

S3 File qPCR data. Used for Fig 4.
(XLSX)

S4 File Data from quantitative study of heterocyst-specific YFP fluorescence. (XLSX)
