# Supplementary figures and images for: Design and characterization of a synthetic minimal promoter for heterocyst-specific expression in filamentous cyanobacteria
Source: PLoS One. 2018 Sep 11;13(9):e0203898. doi: 10.1371/journal.pone.0203898 (PMC6133370; doi:10.1371/journal.pone.0203898)

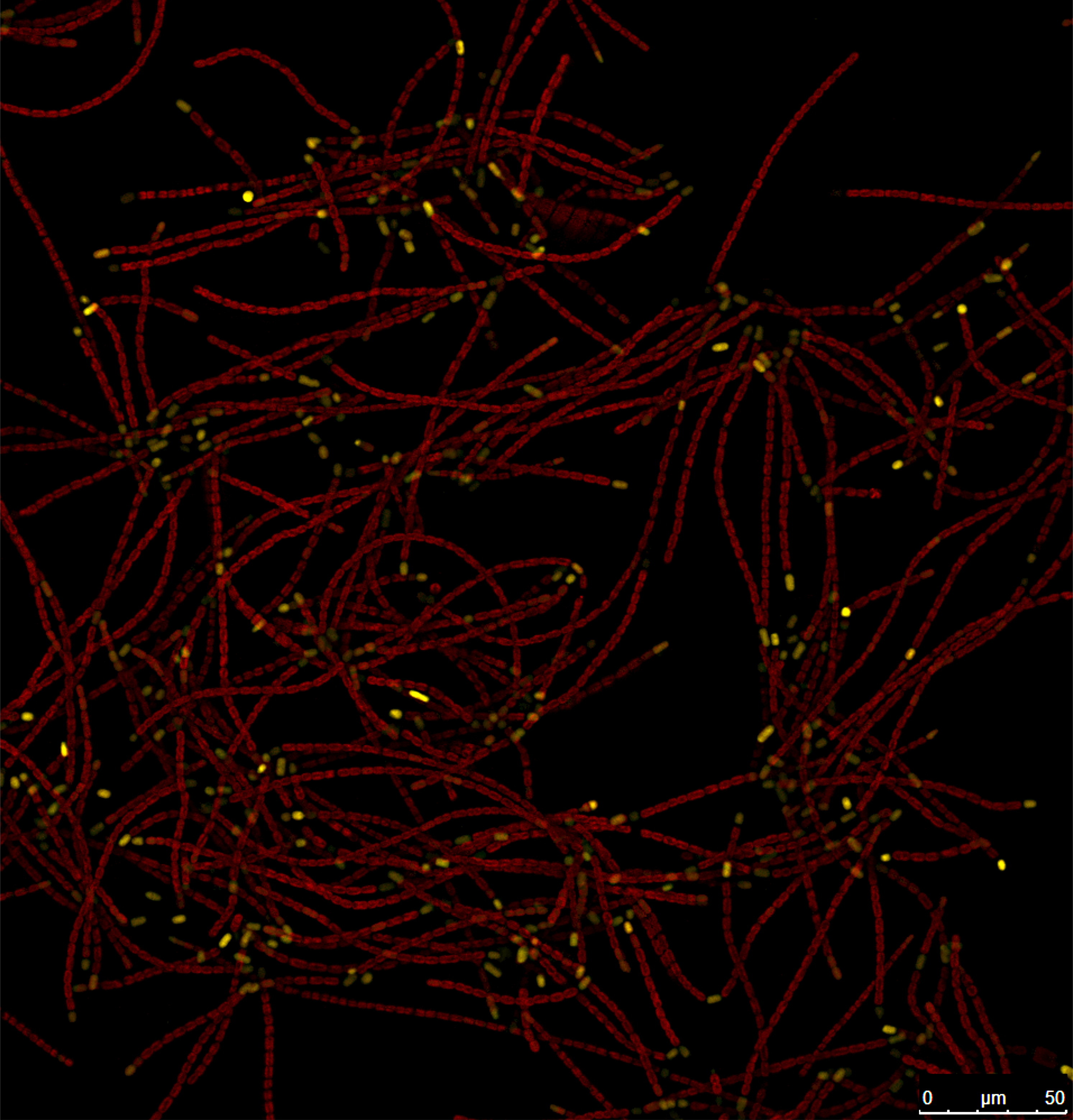

Supplement: S2 Fig — 3D-projection of a Z-stack containing 91 slices of a 30μm deep sample volume. Fluorescence images from YFP channel (530-540nm) and autofluorescence channel (600-700nm) are overlaid. (TIF) [file pone.0203898.s002.tif]

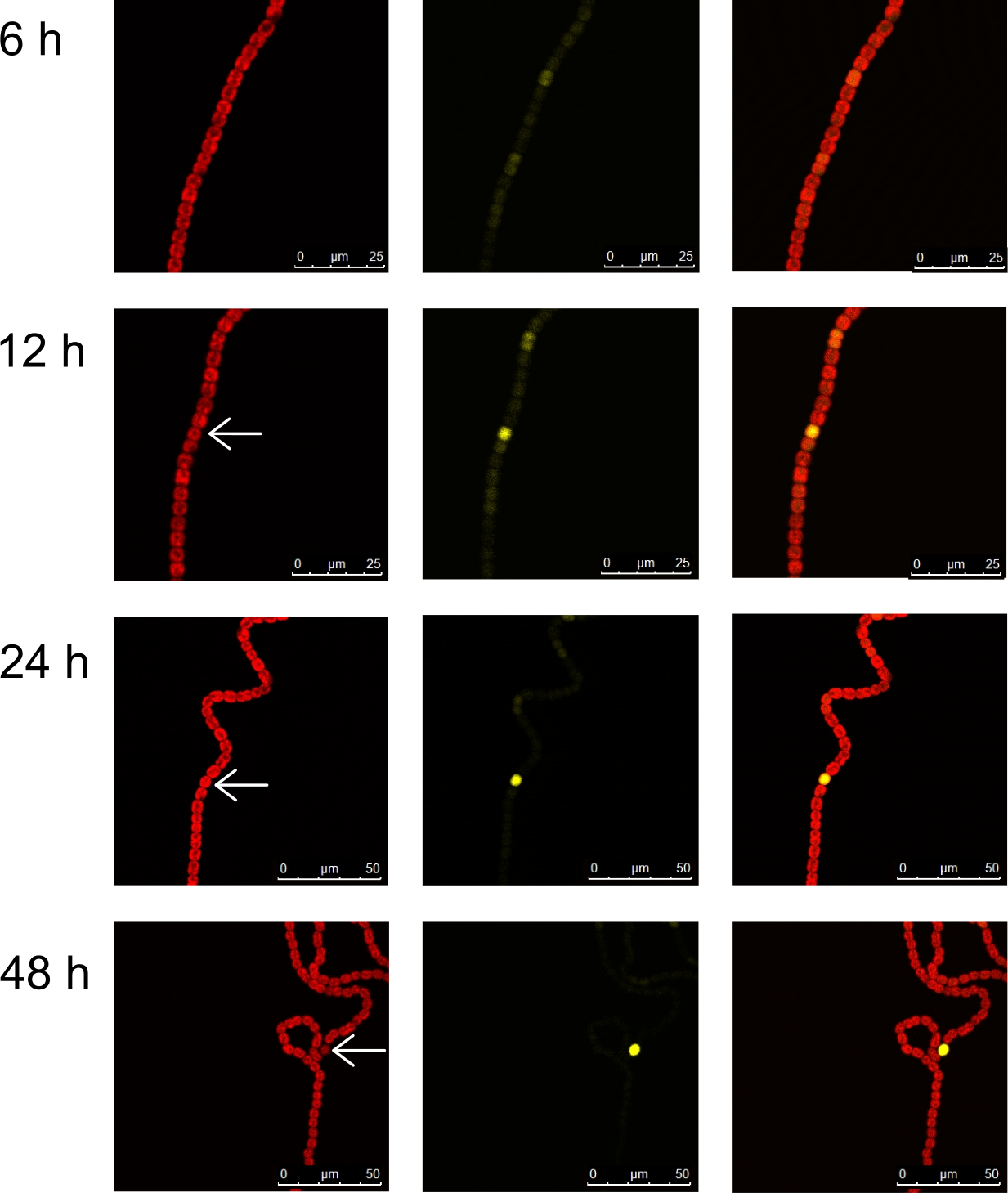

Supplement: S3 Fig — Confocal fluorescence images of a representative filament of the SynDIF strain at 6, 12, 24 and 48 h after nitrogen deprivation immobilized on agar plate. Autofluorescence (600-700nm) is shown in red, YFP fluorescence (530-540nm) in yellow. Rightmost column shows overlaid images. In the autofluorescence images, the arrows indicate a developing heterocyst. (TIF) [file pone.0203898.s003.tif]
