# Supplementary material for: Design and characterization of a synthetic minimal promoter for heterocyst-specific expression in filamentous cyanobacteria
Source: PLoS One. 2018 Sep 11;13(9):e0203898. doi: 10.1371/journal.pone.0203898 (PMC6133370; doi:10.1371/journal.pone.0203898)
Supplement: S1 File — (DOCX) [file pone.0203898.s004.docx]

**S1 File. Investigation of YFP fluorescence in identified heterocysts of the SynDIF and UESynDIF *Nostoc punctiforme* strains.**

Using fluorescence confocal microscopy, we identified twenty heterocyst each, from 3 independent cultivations of the SynDIF (SynDIF 1-3) and UESynDIF (UESynDIF 1-3) strains, by their characteristic morphology and reduced autofluorescence. The identified heterocysts were investigated for YFP fluorescence (530-540nm). If YFP fluorescence was clearly visible, while the 5 neighboring cells on each side showed no detectible fluorescence compared to WT strain vegetative cells, the heterocyst was counted as fluorescing. Results are presented in the table below.

|  | **Investigated heterocysts** | **Fluorescing heterocysts** | **Percent fluorescing** |
| --- | --- | --- | --- |
| **SynDIF 1** | 20 | 20 | 100 |
| **SynDIF 2** | 20 | 20 | 100 |
| **SynDIF 3** | 20 | 20 | 100 |
| **average** | **-** | **20/20** | **100%** |
|  |  |  |  |
| **UESynDIF 1** | 20 | 18 | 90 |
| **UESynDIF 2** | 20 | 19 | 95 |
| **UESynDIF 3** | 20 | 18 | 90 |
| **average** | **-** | **18.3/20** | **91.7%** |
