# Supplementary material for: Design and characterization of a synthetic minimal promoter for heterocyst-specific expression in filamentous cyanobacteria
Source: PLoS One. 2018 Sep 11;13(9):e0203898. doi: 10.1371/journal.pone.0203898 (PMC6133370; doi:10.1371/journal.pone.0203898)
Supplement: S2 File — Full sequences of the promoter-RBS designs synthesized for this study. (DOCX) [file pone.0203898.s005.docx]

**S2 File. Supplementary File 2**

Full sequences of the promoter-RBS designs synthesized for this study.

P_synDIF_

ACGCATTGTTTTTCCGGAGATAGGTAGATAGTTTAGAAAAATTTAATATAGGATCCTAGTGGAGGT

P_UEsynDIF_

TGTTCATAAATAACCAGCATAATCGTGCAGATTCATCCGGAGATAGGTAGATAGTTTAGAAAAATTTAATATAGGATCCTAGTGGAGGT

*Legend*

Spacer/insulating regions

DIF1 motif

Extended -10

TSS

RBS*

Native upstream element
